# Diurnal Ruminal pH and Temperature Patterns of Steers Fed Corn or Barley-Based Finishing Diets

**DOI:** 10.3390/ani11102809

**Published:** 2021-09-27

**Authors:** Hannah M. DelCurto-Wyffels, Julia M. Dafoe, Cory T. Parsons, Darrin L. Boss, Timothy DelCurto, Samuel A. Wyffels, Megan L. Van Emon, Janice G. P. Bowman

**Affiliations:** 1 Department of Animal and Range Sciences, Montana State University, Bozeman, MT 59717, USA; timothy.delcurto@montana.edu (T.D.); megan.vanemon@montana.edu (M.L.V.E.); jbowman@montana.edu (J.G.P.B.); 2 Northern Agricultural Research Center, Montana State University, Havre, MT 59501, USA; jdafoe@montana.edu (J.M.D.); Cory.Parsons2@chsinc.com (C.T.P.); dboss@montana.edu (D.L.B.); samwyffels@montana.edu (S.A.W.)

**Keywords:** barley, corn, intake, ruminal pH, ruminal temperature, steer

## Abstract

**Simple Summary:**

Cattle in feedlots are typically fed diets with a high proportion of cereal grains. While feeding high-energy grain-based diets is advantageous for growth and performance, it can also contribute to an increased likelihood of metabolic issues. Different feedstuffs have unique digestive utilization, which may lead to different cattle performance outcomes. Barley is fermented to a greater extent in the rumen, compared to corn, and can lead to an increased likelihood of digestive disorders. To further our understanding of the use of barley and corn in cattle feedlot diets, we evaluated the effect of diets on ruminal pH, temperature and feed intake events using continuous rumen monitoring technology. While mean ruminal pH was not different between corn or barley-fed steers, barley-fed steers had greater ruminal pH change throughout a 24 h period. Barley-fed steers also exhibited greater variation in ruminal pH. Additionally, intake patterns were different between corn- and barley-fed steers in which corn-fed steers consumed more feed the first 6 h directly after feeding while barley-fed steers consumed more feed later in the day. Presumably these intake patterns could be influenced by differences in the diurnal patterns of ruminal pH between corn and barley. By evaluating ruminal dynamics on a diurnal scale, we will enhance our understanding of utilization of different feedstuffs in beef feedlot diets.

**Abstract:**

This study evaluated the effects of corn or barley finishing diets on ruminal pH and temperature and their relationship to feed intake events using continuous reticulorumen monitoring of feedlot steers. Average daily ruminal pH and temperature were not impacted (*p* ≥ 0.17) by diet. However, diet did affect daily variation of ruminal pH and temperature (*p* < 0.01). Average hourly ruminal pH displayed a diet by hour post-feeding interaction (*p* < 0.01), where barley-fed steers had greater (*p* < 0.01) ruminal pH than corn-fed steers at 0, 1, 18, 19, 20, 21, 22 and 23 h post feeding, but had lower (*p* ≤ 0.05) ruminal pH than corn-fed steers at 6, 7, and 8 h post-feeding. Variation in ruminal pH hour post-feeding also displayed a diet by hour post-feeding interaction (*p* < 0.01), where barley-fed steers had greater (*p* ≤ 0.03) variation in ruminal pH at hours 1–17 post-feeding but did not differ (*p* ≥ 0.16) at 0, 18, 19, 20, 21, 22 and 23 h post-feeding. Additionally, average hourly ruminal temperature exhibited a diet by hour post-feeding interaction (*p* < 0.01). In summary, basal grain interacted with time post-feeding influencing ruminal pH and temperature in feedlot steers.

## 1. Introduction

Feedlot cattle are often fed a diet with a high proportion of cereal grains to meet the energy requirements necessary for targeted growth and performance. Numerous grains may be utilized in cattle feedlot rations. In the United States, corn is the most common grain ingredient in feedlots [1]. However, barley is more adapted to the growing conditions of northern regions and, as a result, is a common feed grain in finishing rations in Canada and the Pacific Northwest [2].

While feeding high concentrate-based rations of rapidly fermentable carbohydrates favors growth and performance, it does come with the challenge of increased likelihood of metabolic disorders such as acidosis [3]. Digestive disorders account for approximately 25% to 33% of deaths in the feedlot and can have a marked impact on cattle health and efficiency of production [4]. The grain source used in feedlot rations can have an influence on both rumen environment and function [5]. In particular, barley starch is more rapidly fermented and digested to a greater extent in the rumen compared to corn [6,7]. Thus, the use of barley in finishing rations has been criticized due to the increased likelihood of metabolic disorders and reduced performance [3,5].

In an ideal rumen environment for grain-fed cattle, ruminal fermentation is stable and mean ruminal pH is generally greater than 5.5, often ranging from 5.8 to 6.5 [8]. Typically, ruminal pH of grain-fed cattle follows a diurnal pattern in which ruminal pH is often high before the morning feeding and declines after feeding through peak fermentation [9]. However, the extent of ruminal pH decline is dependent on the size and fermentability of the meal [9,10]. Ruminal pH is a critical factor for rumen function due to the impact of pH on microbial populations, products of fermentation, as well as the physiological function of the rumen including motility and absorption [8,11]. When ruminal pH drops below 5.6 there is often a shift in microbial populations toward lactic acid production, which will continue to reduce ruminal pH, resulting in acidosis [8]. Recent research has demonstrated that low ruminal pH is also associated with increased ruminal temperature [12,13], establishing an importance for monitoring rumen temperature for determining animal health and early detection of disease [14].

Diet composition and ruminal microbial populations play a large role in digestion and therefore, the efficiency of cattle performance [8]. The digestive characteristics of barley and corn grains could potentially influence ruminal environment and cattle dry matter intake. However, the effects of intake on gastrointestinal function in cattle are less understood [15]. Moreover, information relating individual intake to ruminal environment of animals on barley- or corn-based diets is limited. Therefore, the objective of this study was to evaluate the effects of corn or barley finishing diets on ruminal pH, temperature and feed intake events of feedlot steers using continuous rumen monitoring.

## 2. Materials and Methods

All animals used in this study were provided by the Northern Agricultural Research Center, a unit of the Montana Agricultural Experiment Station, (Havre, MT, USA; Montana; 48.5500° N, 109.6841° W). Experimental design and procedures were approved by the Agriculture Animal Care and Use Committee of Montana State University (#2016-AA26).

Experimental design and diets have been published previously in DelCurto-Wyffels et al. [16]. Briefly, Angus-based yearling steer calves were fed in a feedlot study from 27 February 2017, to 12 June 2017 (105 days; 427.3 ± 3.7 kg; *n* = 48) in year 1, and 26 February 2018, to 11 June 2018 (105 days; 406.8 ± 3.4 kg; *n* = 47) in year 2. All steers were implanted at the initiation of this study (Synovex One Feedlot Implant; Zoetis, Parsippany-Troy Hills, NJ, USA). Upon entry to the feedlot, steers were stratified by body weight (BW) and, within stratum, randomly assigned to one of two primary basal grain dietary treatments, including (1) Grade 2 feed corn or (2) Hockett barley. Hockett is a two-row malting barley that is very stable under dryland conditions and often used as a livestock feed source when malting parameters are not met [17]. Both barley and corn grains were dry-rolled and provided as a total mixed ration (Table 1). The diets averaged 10.28% crude protein and 0.24 Mcal∙kg^−1^ net energy gain. Prior to the data collection period, steers were acclimated to their respective diet for 14 days. Steers were fed once daily at 0800 and rations were provided to attain maximum individual intake without excessive wastage. Diets were increased by 0.23 kg per head after clean bunks had been present by midday for two consecutive days. Feed refusals were weighed and removed weekly. All animals had ad libitum access to water for the entirety of the trial.

Steers were fitted with an electronic identification ear tag and were adapted to a GrowSafe system (GrowSafe Systems Ltd., Airdrie, AB, Canada) for 14 days prior to the start of this study. A total of 24 GrowSafe electronic feed bunks (12 per treatment; 1 per 2 steers) were used in this study, each equipped with an antenna to detect animal presence. Neck bars on feed bunks only allowed for one animal entry to a bunk at a time. Individual animal intake was continuously recorded with load cells measurements via wireless transfer to a data-acquisition computer. The system was monitored daily for unaccounted feed balance. Whenever unaccounted feed disappearance was greater than 5% the GrowSafe system deemed the 24 h data collection period as failed. In our study, 8.54% and 10.92% of the dry matter intake data failed in year 1 and 2, respectively. Previous research with GrowSafe technology has suggested that the accuracy of dry matter intake was not impacted when up to 30% of the data were missing [18].

To determine ruminal pH and temperature, an indwelling wireless data transmitting system (SmaXtec^®^ Animal Care GmbH, Graz, Austria) was used [19]. A SmaXtec bolus was administered to 12 steers per treatment group for each year of this study. The pH probes were calibrated using pH 4 and pH 7 buffer solutions. According to directions of the manufacturer, the boluses were inserted into the reticulorumen of the steers at the initiation of this study. SmaXtec animal care technology^®^ enables the continuous real-time display of data such as ruminal pH and temperature. The data were read every 10 min throughout the entirety of this study and all data were obtained by smaXtec messenger^®^ computer software.

Average daily intake and intake behavior are presented in a companion study [16]. GrowSafe data were used to calculate intake for each individual steer each hour post-feeding on a daily basis. The length of time between intake readings that constitute a new intake event was predetermined as 300 s [20,21,22]. Hourly intake and intake event data were then paired with the ruminal pH and temperature readings for each individual steer for the duration of both study years (Table S1). Daily and hourly variation in ruminal pH and temperature, measured as coefficient of variation (CV, %), was based on SmaXtec bolus data for each individual. Data were then used to evaluate ruminal pH and temperature change post-feeding and post-intake event and the variation of ruminal pH and temperature post-feeding.

All statistical analyses were performed in R 4.0.3 [23]. Average daily ruminal pH, temperature, and coefficient of variation (CV, %) of daily ruminal pH and temperature were analyzed using ANOVA (the car package; [24]) with a generalized linear mixed model (the lme4 package; [25]) including diet as the fixed effect, with year and individual steer as random intercepts. Prior to evaluating the effects of diet on diurnal ruminal pH and temperature change, we conducted a Pearson’s correlation test to evaluate the relationship between ruminal pH and temperature. However, preliminary results suggest that there was little relationship between ruminal pH and temperature for both barley- and corn-fed steers (r^2^ = −0.02 and −0.01, respectively). Therefore, ruminal pH and temperature were both included in the final analysis and analyzed independently. Post-feeding hourly intake and ruminal pH, pH CV, ruminal temperature and temperature CV were analyzed using ANOVA (the car package; [24]) with a generalized linear mixed model (the lme4 package; [25]) including diet, hour post-feeding and the interaction of diet and hour post-feeding as fixed effects, with year and individual steer as random intercepts. Ruminal pH and temperature post-feed intake event were analyzed using ANOVA (the car package; [24] with a generalized linear mixed model (the lme4 package; [25] including fixed effects of diet, minutes post-intake event and the interaction of diet and minutes post-intake event and random intercepts of year and individual steer. To account for autocorrelation of repeated measurements, individual steer was used as a random intercept in all analyses. To satisfy assumptions of normality and homogeneity of variance, data were plotted and log transformed if needed. Significance was considered at an alpha ≤ 0.05 and tendencies were considered at an alpha ≤ 0.10. Mean separation was conducted using the Tukey method when *p* < 0.05 (the emmeans package; [26]). Experimental unit was individual steer.

## 3. Results

**Figure 1:**
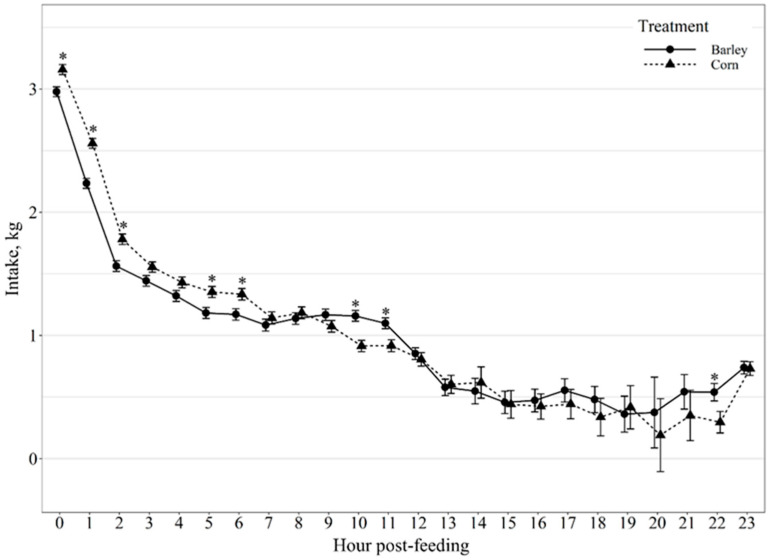
Diurnal dry matter intake patterns of beef steers fed once daily (0800) barley- or corn-based feedlot diets. Intake was influenced by diet × hour post-feeding interaction (*p* < 0.01) and differences (*p* ≤ 0.05) within hour post-feeding are denoted by *.

Average daily ruminal pH and temperature were not impacted (*p* ≥ 0.17; Table 2) by diet. However, diet did affect daily variation of ruminal pH and temperature (*p* < 0.01), where barley-fed steers exhibited 1.74 and 0.25% greater daily variation of pH and temperature, respectively, compared to corn-fed steers. Average hourly intake (kg) post-feeding displayed a diet by hour post-feeding interaction (*p* < 0.01; Figure 1), where corn-fed steers consumed more feed (*p* ≤ 0.01) than barley-fed steers at 0, 1, 2, 5, and 6 h post-feeding and tended (*p* ≤ 0.08) to consume more feed than barley-fed steers at hours 3 and 4 post-feeding. However, barley-fed steers consumed more feed (*p* ≤ 0.03) than corn-fed steers at 10, 11 and 22 h post-feeding.

**Figure 2:**
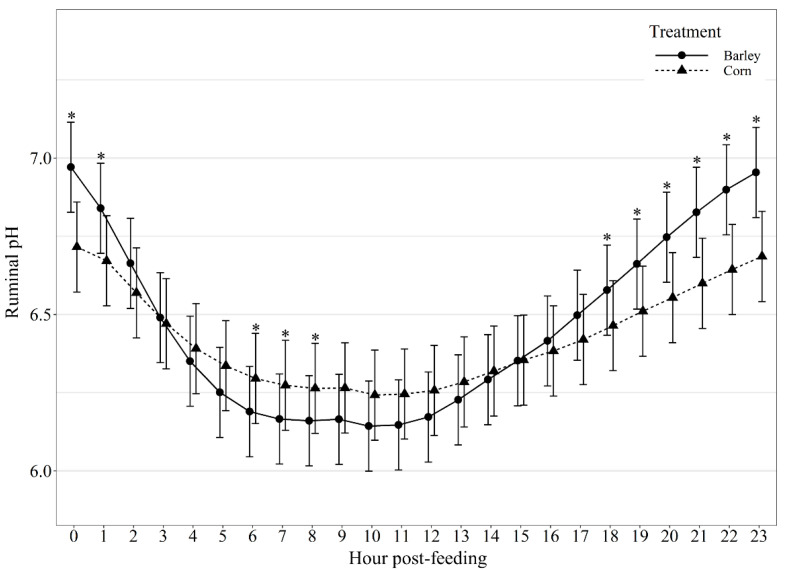
Diurnal pH patterns of beef steers fed once daily (0800) barley- or corn-based feedlot diets. Ruminal pH was influenced by diet × hour post-feeding interaction (*p* < 0.01) and differences (*p* ≤ 0.05) within hour post-feeding are denoted by *.

**Figure 3:**
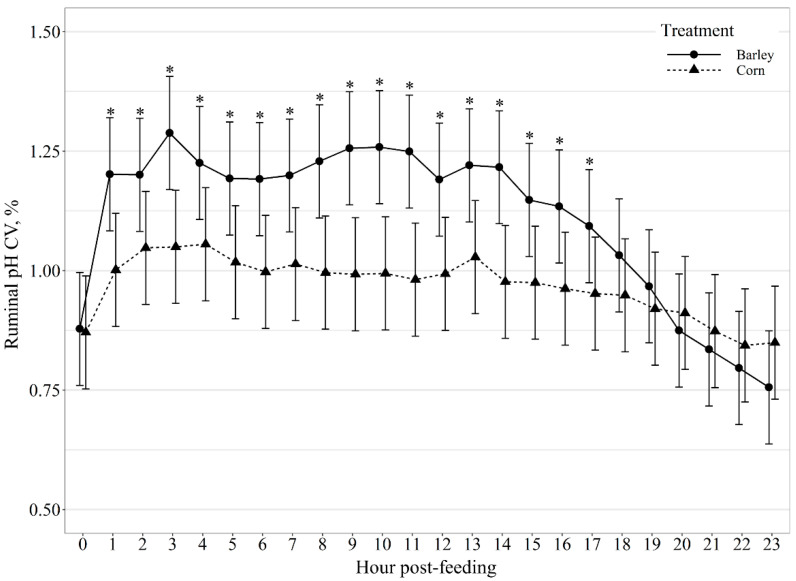
Diurnal pH coefficient of variation (CV, %) patterns of beef steers fed once daily (0800) barley- or corn-based feedlot diets. Ruminal pH CV was influenced by diet × hour post-feeding interaction (*p* < 0.01) and differences (*p* ≤ 0.05) within hour post-feeding are denoted by *.

Average hourly ruminal pH displayed a diet by hour post-feeding interaction (*p* < 0.01; Figure 2). Barley-fed steers had greater (*p* < 0.01) ruminal pH than corn-fed steers at 0, 1, 18, 19, 20, 21, 22, and 23 h post-feeding and tended (*p* = 0.07) to be greater than corn-fed steers 2 h post-feeding, but had lower (*p* ≤ 0.05) ruminal pH than corn-fed steers at 6, 7, and 8 h post-feeding and tended (*p* = 0.06) to be lower than corn-fed steers at 9, 10, and 11 h post-feeding. Additionally, variation in ruminal pH hour post-feeding also displayed a diet by hour post-feeding interaction (*p* < 0.01; Figure 3), where barley-fed steers had greater (*p* ≤ 0.03) variation in ruminal pH at hours 1–17 post-feeding but did not differ from corn-fed steers (*p* ≥ 0.16) at 0, 18, 19, 20, 21, 22, and 23 h post-feeding. Suggesting that barley-fed steers had greater decline in pH post-feeding and greater variation in ruminal pH than corn-fed steers.

**Figure 4:**
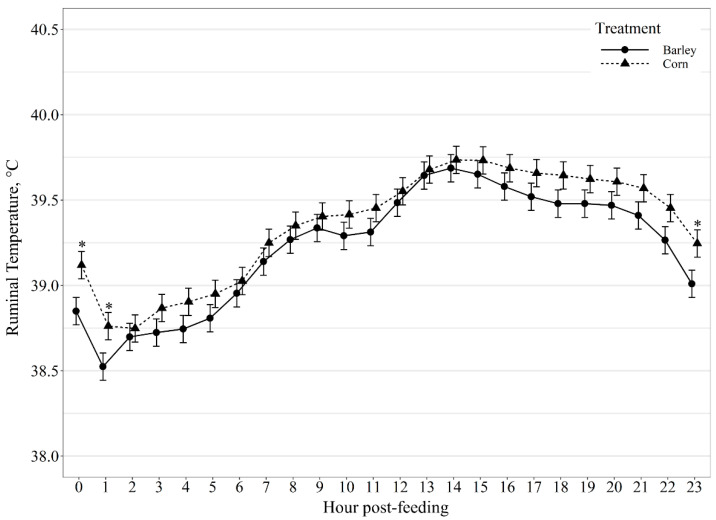
Diurnal ruminal temperature (°C) patterns of beef steers fed once daily (0800) barley- or corn-based feedlot diets. Ruminal temperature was influenced by diet × hour post-feeding interaction (*p* < 0.01) and differences (*p* ≤ 0.05) within hour post-feeding are denoted by *.

**Figure 5:**
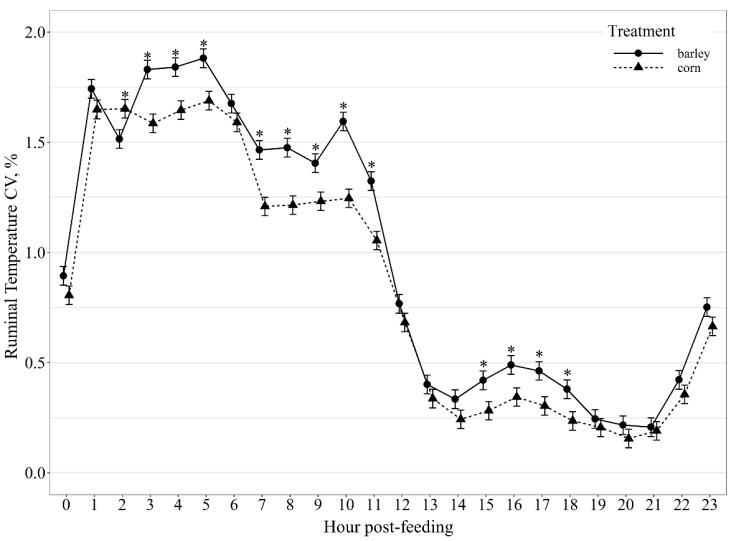
Diurnal ruminal temperature coefficient of variation (CV, %) patterns of beef steers fed once daily (0800) barley- or corn-based feedlot diets. Ruminal temperature CV was influenced by diet × hour post-feeding interaction (*p* < 0.01) and differences (*p* ≤ 0.05) within hour post-feeding are denoted by *.

Average hourly ruminal temperature exhibited a diet by hour post-feeding interaction (*p* < 0.01; Figure 4), with corn-fed steers having greater (*p* ≤ 0.01) ruminal temperature at 0, 1, and 23 h and tended (*p* ≤ 0.10) to have greater ruminal temperature at 4, 18, 21 and 22 h post-feeding. Variation in ruminal temperature hour post-feeding also displayed a diet by hour post-feeding interaction (*p* < 0.01; Figure 5), where corn-fed steers had greater (*p* = 0.02) variation in ruminal temperature at 2 h post-feeding than barley-fed steers; however, barley-fed steers had greater (*p* ≤ 0.02) variation in ruminal temperature than corn-fed steers at 3, 4, 5, 7, 8, 9, 10, 11, 15, 16, 17 and 18 h post-feeding.

**Figure 6:**
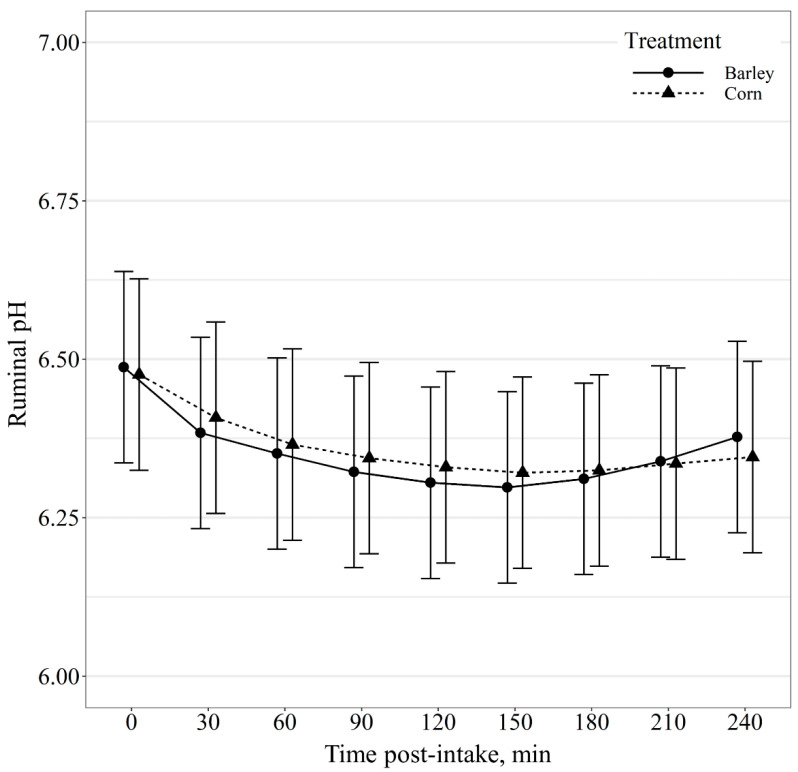
Ruminal pH change as a function of time (min) relative to intake event of beef steers provided barley- or corn-based feedlot diets. Ruminal pH was influenced by a diet × minute post-intake event interaction (*p* < 0.01); however, no differences (*p* ≥ 0.63) were observed between diets within post-intake event time periods.

**Figure 7:**
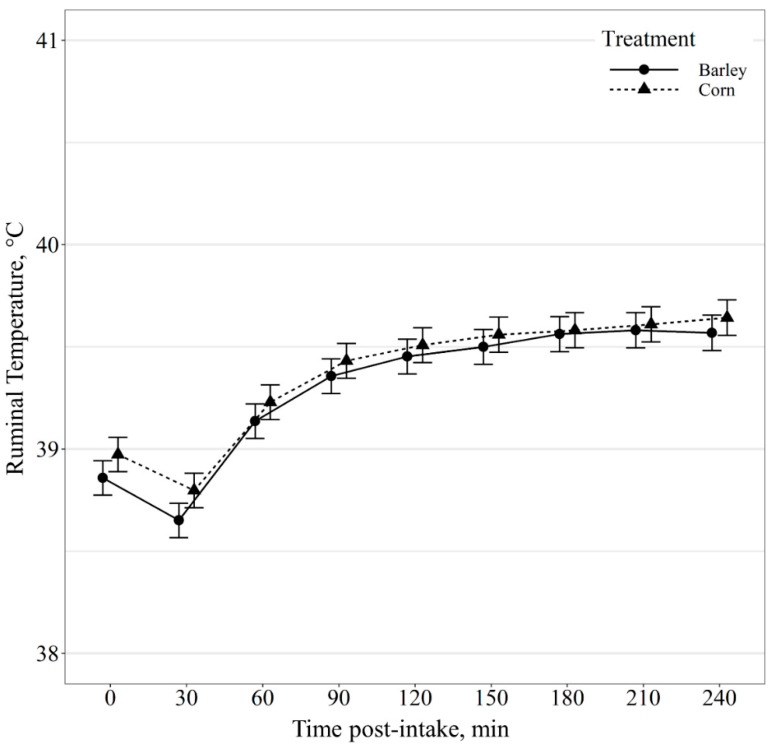
Ruminal temperature (°C) change as a function of time (min) relative to intake event of beef steers provided barley- or corn-based feedlot diets. Ruminal temperature was influenced by a diet × minute post-intake event interaction (*p* < 0.01); however, no differences (*p* ≥ 0.11) were observed between diets within post-intake event time periods.

Ruminal pH post-intake event displayed a diet by minute post-intake event interaction (*p* < 0.01; Figure 6); however, post hoc mean separation displayed no diet effects (*p* ≥ 0.63) on ruminal pH within minute post-intake event. Similarly, ruminal temperature post-intake event also exhibited a diet by minute post-intake event interaction (*p* < 0.01; Figure 7); however, post hoc means separation displayed no diet effects (*p* ≥ 0.11) on ruminal temperature within minute post-intake event.

## 4. Discussion

In the United States, the majority of cattle in feedlots are fed a grain-based diet [1]. The feedstuff utilized can impact cattle performance, rumen dynamics and digestive efficiency. In addition, the intake of fermentable carbohydrates coupled with the rate of fermentation can impact ruminal pH. Barley and corn are two commonly used feeds for cattle; however, the digestive utilization of each grain can be quite different. Generally, corn has a greater starch content than barley and the starch is more likely to escape ruminal digestion and be absorbed in the small intestine [6]. Because starch digestion in the rumen is greater for barley than corn it has been demonstrated that cows fed diets containing barley tend to have a ruminal pH 0.2 units lower than cows fed corn when fed the same amount of forage fiber [10]. In our study, we did not observe a difference in daily mean ruminal pH between barley and corn-fed steers and mean ruminal pH for steers fed both diets were well above acidotic conditions. However, nutritionists commonly attribute metabolic disturbances to large daily shifts in feeding behavior and erratic feed intake by cattle [27,28]. The size, number, and frequency of meals can have a large effect on ruminal pH [27] with low ruminal pH further contributing to erratic intake patterns [29]. A companion study evaluating the effect of corn- and barley-based diets on intake behavior and performance using the same steers, demonstrated a tendency for corn-fed steers to consume more feed per day and have greater variation in intake per day than barley-fed steers, but showed no differences in number and frequency of meals [16]. However, the differences in regard to intake patterns were not manifested in average daily ruminal pH.

Generally, ruminal pH is highest just before the morning feeding. After feeding, the pH drops in relationship to fermentation of dietary carbohydrates [10,27]. Although we did not observe a difference in mean daily pH between barley and corn-fed steers, barley-fed steers displayed greater variation in daily ruminal pH and greater variation in ruminal pH for the majority of hours post-feeding (hours 1–17) compared to corn-fed steers. These findings could be due to the difference in starch availability and rate of fermentation between the two feedstuffs. Corn starch is less ruminally digestible [6] with up to 30% of corn starch escaping ruminal fermentation. Corn also has a slower rate of in situ dry matter and starch disappearance [30]. The slower rate of ruminal digestion of corn-based diets reduces the risk of low reticulorumen pH and may allow for the more stable diurnal pH observed in our study.

Previous research has reported a strong correlation between feed intake of feedlot cattle and the lowest daily ruminal pH on the previous day [31]. Yang et al. [32] observed that ruminal pH was higher in cows fed a corn-based diet compared to barley throughout the early portions of the day post-feeding. However, the magnitude of difference in ruminal pH between barley and corn-based diets were relatively small [32,33]. In our study, both corn- and barley-fed steers exhibited the majority of daily intake the first 12 h post-feeding, with corn-fed steers consuming or tending to consume more feed than barley-fed steers within the first 6 h post-feeding; however, barley-fed steers consumed more feed than corn-fed steers at hour 10 and 11 post-feeding and immediately prior to the next feeding event at 22 h. Additionally, we found a diurnal difference in ruminal pH between barley and corn-fed steers in which ruminal pH was higher in barley-fed steers 0 and 1 h post-feeding, as well as, 18–23 h post-feeding and lower than corn-fed cattle at hour 6, 7, and 8 post-feeding. Thus, the ruminal pH for barley-fed steers had greater daily ruminal pH declines compared to corn-fed steers. Previous research has found that cattle consuming high levels of barley alter feeding behavior in a manner that reduces the risk of acidosis [34]. These findings suggest that animals may adjust their intake when pH is low, presumably in an attempt to limit consumption of fermentable carbohydrates and restore pH conditions to a more comfortable level [27]. This may explain why diet had an effect on diurnal ruminal pH patterns but was not a factor influencing ruminal pH post-intake event out to 240 min.

Previous research has demonstrated ruminal temperature to be a reliable measure of body temperature in both dairy cows and beef steers [14,35]. Thus, monitoring ruminal temperature could aid in detecting adverse health events in cattle [14]. Additionally, a correlation has been observed between rumen temperature and pH [12,36]. The strength of this relationship, however, appeared to be influenced by diet composition and individual animal variability [36]. In the current study, rumen temperature responded similarly in both barley and corn-fed cattle apart from the hour before and after feeding. There was no difference in ruminal temperature post-intake events between barley and corn-based diets. However, for steers fed barley- or corn-based diets, rumen temperatures declined 30 min post-intake event. Rumen temperatures are impacted by other factors such as water intake [37,38]. Presumably, the reduced temperatures observed 30 min post-intake could be due to cattle consuming water after feeding. Previous research has predicted watering events based on ruminal temperature change recorded with rumen boluses; however, this comes with a large margin of error [39]. Therefore, the relationship of rumen pH and temperature with water intake and intake behavior warrants further investigation.

## 5. Conclusions

Barley and corn are two commonly used grain sources in beef cattle diets. However, digestive utilization of each feedstuff and subsequent rumen environment of cattle fed barley- or corn-based diets can be markedly different. While mean ruminal pH was not different between corn or barley-fed steers, barley-fed steers had greater ruminal pH change throughout a 24 h period. Additionally, barley-fed steers had greater diurnal variation in ruminal pH up to hour 17 post-feeding. These findings are likely due to differences in starch content and rate of fermentation of the grains. Intake patterns were also unique in corn- and barley-fed steers in which corn-fed steers consumed more feed the first 6 h directly after feeding. Barley-fed steers, however, consumed more feed later in the day. Presumably these intake patterns could be influenced by ruminal pH. Our research suggests that evaluating intake and ruminal fermentation characteristics needs to be conducted at a diurnal scale to optimize beef cattle performance and efficiency.

## Figures and Tables

**Table 1:** Ingredient composition of finishing diets containing corn or Hockett barley as basal grains.

	Barley	Corn
Ingredient		
Corn, %	−	80.00
Barley, %	80.00	−
Barley straw, %	12.00	12.00
Canola oil, %	3.00	3.00
Supplement, % ^1^	5.00	5.00

^1^ The supplement composition was formulated based on initial basal grain nutrient analysis and designed to make diets similar in crude protein, minerals and vitamins.

**Table 2:** Daily dry matter intake, ruminal pH and temperature of steers consuming diets containing corn or Hockett barley as basal grains at the Northern Agricultural Research Center, Havre, MT, USA.

Item	Barley	Corn	SEM ^1^	*p*-Value
Average daily intake, kg ^2^	11.30	11.72	0.52	0.06
Average daily ruminal pH	6.46	6.43	0.14	0.46
Daily ruminal pH CV, % ^3^	5.63	3.89	0.14	<0.01
Average daily ruminal temperature, °C	39.22	39.35	0.08	0.17
Daily ruminal temperature CV, %	2.37	2.12	0.10	<0.01

^1^ SEM = standard error of the means. ^2^ Average daily intake (kg) originally presented in DelCurto-Wyffels [16]. ^3^ CV = coefficient of variation.

## Data Availability

The data presented in this study are available in Table S1.

## Supplementary Materials

The following are available online at https://www.mdpi.com/article/10.3390/ani11102809/s1. Table S1: Animal Intake and Ruminal pH and Temperature Data.
